# Biodegradability of oily wastewater using rotating biological contactor combined with an external membrane

**DOI:** 10.1186/s40201-014-0117-3

**Published:** 2014-09-02

**Authors:** Mahdieh Safa, Iran Alemzadeh, Manouchehr Vossoughi

**Affiliations:** Chemical and petroleum engineering department, Sharif University of Technology, Azadi St, Tehran, Iran

**Keywords:** Biological treatment, Hybrid membrane bioreactor, Oily wastewater, Rotating biological contractor

## Abstract

**Background:**

A novel implementation of a hybrid membrane bioreactor (HMBR) has been studied in this paper. It is utilized as combination of rotating biological contractor (RBC) and an external membrane, as a new biological system for oily wastewater treatment.

**Methods:**

Chemical oxygen demand (COD) and total petroleum hydrocarbon (TPH) as factors of Biodegradability has been evaluated. They are both compared together for different hydraulic retention times (HRTs) and petroleum pollution concentrations in RBC and HMBR. The ratio of TPH to COD of Molasses has been varied between 0.2 to 0.8 at two HRTs of 18 and 24 hours while the temperature, pH and dissolved oxygen were kept in the range of 20-25°C, 6.5-7.5, and 2-3.5 mg/l, respectively.

**Results:**

The best TPH removal efficiency (99%) was observed in TPH/COD = 0.6 and HRT = 24 hr in HMBR and Removal efficiency was decreased in the ratios above 0.6 in both bioreactors.

**Conclusions:**

The experimental results showed that HMBR had higher treatment efficiency than RBC at all ratios and HRTs.

## Background

Nowadays, one of the major environmental problems is the oily wastewaters produced by industries, particularly by refineries. Disposal of oily wastewaters into the environment can result in environmental pollutions and serious damages to the ecosystem. Since conventional treatment processes are not sufficient to achieve the water quality requirements, advanced treatment processes are required [1].

The HMBR is an advanced technology which traditionally combines activated sludge as a suspended growth system with microfiltration (MF) or ultra filtration (UF) membrane [2]. This process has now become an attractive choice for the treatment and reuse of industrial wastewaters such as paper mill; food production; fuel port [3–5] and municipal wastewaters [6,7]. The HMBR process has been proved to have many advantages in comparison to conventional biological processes such as small footprint size of the treatment unit, reduced sludge production, complete retention of solids and flexibility of operation [8].

The initiative of the present research is substituting the suspended growth system with the attached growth system. Therefore, RBC (plus Kaldnes media) as an attached growth system was coupled with external UF membrane to treat oily wastewater.

The reason for choosing RBC can be related to many advantages of this reactor in treating wastewaters, particularly oily wastewaters, compared to the active sludge process. Among the advantages, one can include high efficiency of organic matter removal, resistance against organic and hydraulic shock loads and low energy consumption [9].

Experiments were carried out to compare the performance of the RBC and the HMBR in treating the oily wastewaters. After adjusting oil-eating microorganisms with system, the influence of some parameters as HRT, TPH and nutrients concentration on the performance of system were studied. The efficiency of two systems in removal of oily pollutants and organic matters produced by nutrients was also examined and compared.

## Methods

### Physical properties of the system

**Figure 1:**
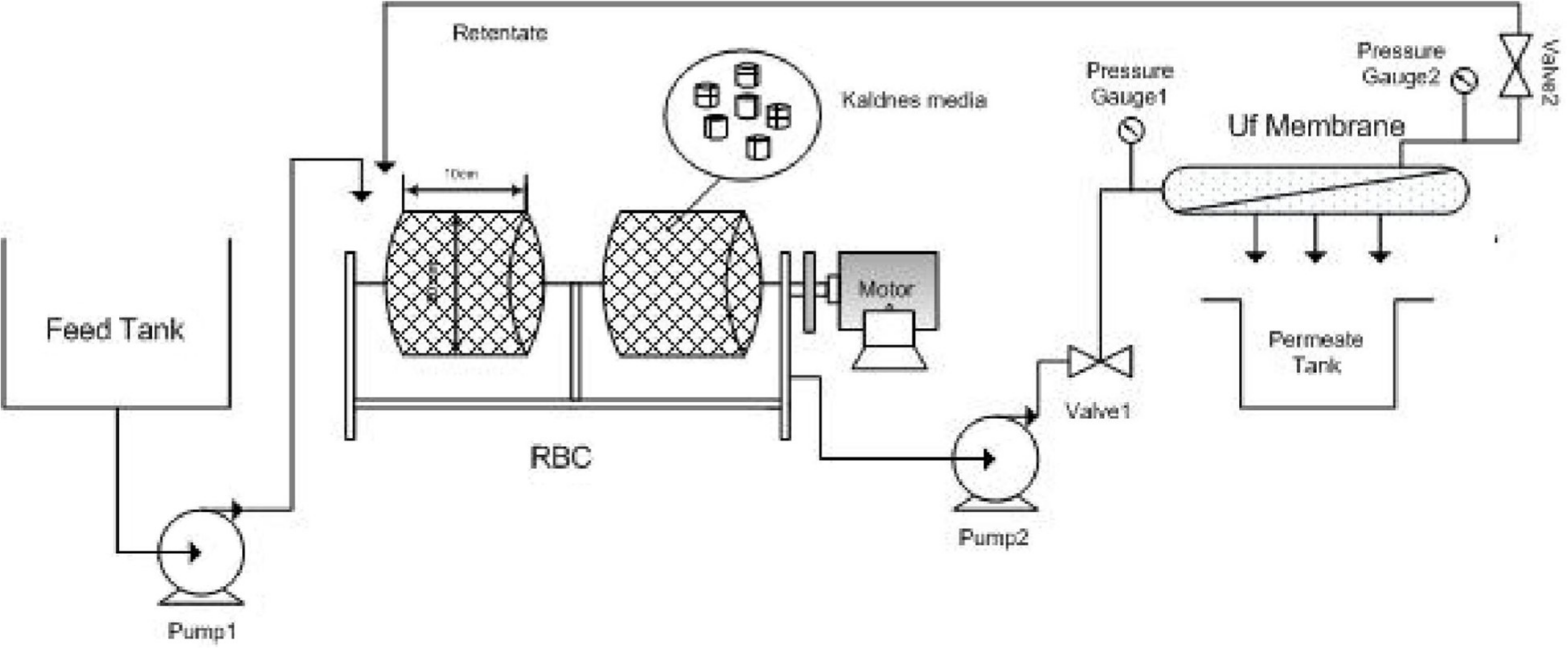
**Overview of the hybrid membrane.**

Figure 1 shows an overview of the hybrid membrane bioreactor. HMBR is a combination of rotating biological contactor (plus Kaldnes media) and an external membrane. Effluent from bioreactor enters the membrane by a centrifugal pump and the sludge remained behind the membrane, which contains microorganisms, is returned to the bioreactor. Samples were collected from influent/effluent of RBC and effluent of the membrane. Physical properties of RBC and the membrane are shown in Tables 1 and 2, respectively.

**Table 1:** **Physical properties of the rotating biological contactor**

**Length**	40 cm	**Width**	27 cm
**Height**	20 cm	**Total surface**	2m^2^
**Total volume**	21.6 lit	**Effective volume**	18 lit
**Cylinder diameter**	20 cm	**Cylinder length**	10 cm
**Stage**	2	**Rotational speed**	10 rpm

**Table 2:** **Physical properties of the membrane**

**Membrane type**	**Ultra filtration**
**Membrane material**	Polymer
**Internal diameter**	1.24 cm
**Effective length**	33 cm
**Surface area**	0.0128 m^2^

### Bioreactor feeding

In this study, the sludge of second settling tank of the activated sludge process in Tehran refinery was used. At the first, RBC was prepared and 90% volume of its cylinders was filled with Kaldnes media. Then, some of the sludge plus some water was poured into the bioreactor so that the mixed liquor suspended solids (MLSS) in bioreactor became 1500 mg/l.

In order to grow and reproduce microorganisms and biofilm formation, the system was set up in batch process with COD = 1000 mg/l so it fed with carbon (molasses), nitrogen (urea) and phosphorus (ammonium phosphate) for 8 weeks. During the process, a combination of crude oil and gasoline with a ratio of 2/1 (petroleum pollutant in this state would have a wide range of hydrocarbons from C14 to C42) was added to the system for more adaption of microorganisms to the petroleum pollutant. Also, 5-15 μl/l of surfactant twin-80 was added to system so that bonds are formed between water and oil molecules. To speed up the microorganisms growth, some minerals were added to the sludge as well [10].

### Experimental process

Once the biofilm with a thickness of about 4 mm was formed, the system was started up as continuous process at a HRT of 24 and 18 hours. The external membrane was connected to RBC. Subsequently, wastewater influent and effluent of RBC and effluent of HMBR were examined daily. These tests included the measurement of COD, MLSS, MLVSS, TPH, TSS, pH, temperature, and dissolved oxygen. All tests were performed according to the standard methods [11]. As time passes, membrane fouling causes such a permeate flux decline that the membrane needs to be refreshed. For this reason, bioreactor was switched off and the membrane was washed with water, NaOH 2%, and HNO_3_ 1% [12].

## Results

### Effect of hydraulic retention time on COD removal

**Figure 2:**
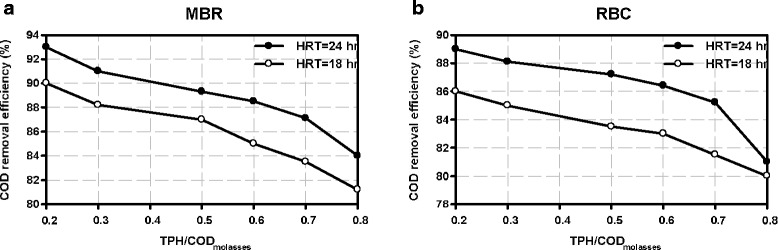
**COD removal efficiency at two HRTs of 18 and 24 hours versus ratios of TPH/COD molasses in (a) MBR and (b) RBC.**

Figure 2 shows the diagram of COD removal efficiency versus different concentrations of TPH at two HRTs (18 & 24 hr) for each reactor.

As shown in Figure 2 increasing the ratios of TPH/COD molasses has led to the reduction of COD removal efficiency in both reactors. This is resulted from the fact that increasing TPH/COD molasses makes microorganisms start to use oily hydrocarbons instead of nutrients produced by molasses.

On the other hand, in order to form bonds between water and oily pollutant molecules, some concentration of surfactant twin-80 was added to the system which helps the absorption of hydrocarbons by microorganisms.

As depicted in Figure 2 when the ratio of TPH/COD was greater than 0.6, the slope of the efficiency decrement was increased. This is due to inhibition caused by aromatics and hydrocarbons in oily wastewater. Furthermore, COD removal efficiency was increased for higher HRT. This is caused by the contact between nutrients and microorganisms for a longer retention time.

### The effect of HRT on TPH removal

**Figure 3:**
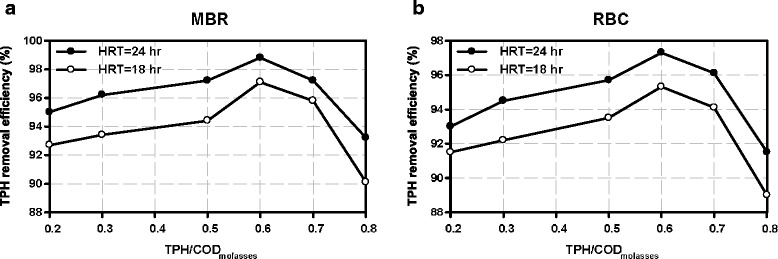
**TPH removal efficiency at two HRTs of 18 and 24 hours versus ratios of TPH/COD molasses in (a) HMBR (b) RBC.**

TPH removal efficiency versus different concentrations of TPH at two HRTs (18 & 24 hr) for each reactor is shown in Figure 3.

As shown in Figure 3 increasing HRT has led to increasing the TPH removal efficiency, because pollutants contacted microorganisms for a long hydraulic retention time.

Increasing the ratio of TPH/COD molasses to 0.6 has led to increasing the TPH removal efficiency in both reactors but when the ratios of TPH/COD molasses was greater than 0.6, the efficiency of both systems in removing the pollutant was reduced. This is due to the fact that the increase in the concentration of hydrocarbons on biofilm distorts the cellular metabolism of microorganisms and prevents them from using carbon molasses for their metabolism and reproduction. This will, in turn, reduce MLSS in system and the potential for removing the pollutant will be significantly reduced. Thus, in treating the oily wastewater in such reactors, it is recommended not to choose the ratio of TPH/COD molasses more than 0.6.

### The effect of various ratios of TPH/COD molasses on TPH removal efficiency

**Figure 4:**
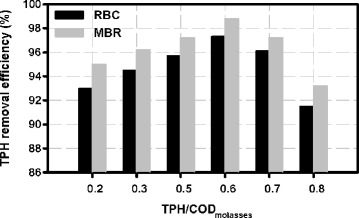
**Comparing TPH removal efficiency in RBC and HMBR in different ratios of TPH/COD molasses at HRT of 24 hours.**

Figure 4 shows the TPH removal efficiency for ratios of TPH/COD molasses at HRT of 24 hours in both reactors.

This comparison shows that TPH removal efficiency for all concentrations of the oily pollutant used in this project has been higher in hybrid membrane than RBC.

### The effect of various ratios of TPH/COD molasses on suspended solids removal efficiency

**Figure 5:**
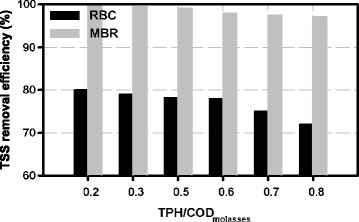
**Comparing the suspended solids' removal efficiency in RBC and hybrid membrane bioreactor for various ratios of TPH/COD at HRT of 24 hours.**

Figure 5 shows the suspended solid's removal efficiency by two reactors at various concentrations of the pollutant.

This comparison shows that as the concentration of the oily pollutant increases, the suspended solids removal efficiency is reduced in both reactors. The effluent suspended solids of the system was increased with increasing oily pollutant concentrations because the bio-film detached from the media due to the toxicity of oily pollutant [13].

Also the diagram shows the higher efficiency of HMBR than the RBC in removing the suspended solids because of the membrane performance.

### Investigating the changes of permeate flux from membrane over time

**Figure 6:**
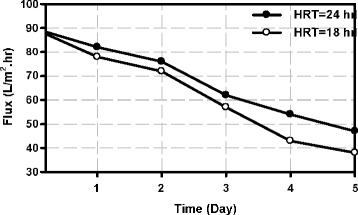
**Permeate flux of membrane versus time.**

Figure 6 shows the changes of permeate flux from membrane in a typical pressure of 1.2 bar.

When the permeate flux of the membrane was about 30 L/m^2^.hr ( it takes 6 days for average MLSS of 3000mg/l and about 5 days for higher concentrations) chemical cleaning of the membrane is performed.

Higher permeate flux of the membrane at HRT of 24 hours than 18 hours proves higher efficiency of removing organic substances and suspended solids and thus reduction of the membrane fouling and higher permeate flux as well.

## Conclusions

In this paper, the behavior of hybrid membrane bioreactor in various loadings of oily pollutant was studied and the results were compared with the time when the rotating biological contactor performs without using a membrane.

The Attached growth bioreactor creates the biofilm on the support media that provide a better treatment efficiency than suspended growth bioreactor due to accumulation of high microbial population in a large surface area. Therefore, better performance can be achieved by combining such a biofilm reactor as RBC with a membrane compared to suspended growth bioreactors as the active sludge in convectional HMBRs.

RBC requires a secondary settling tank which is accomplished by adding a membrane to the system. However, it has a smaller volume than the settling tank and the amount of suspended solids in its effluent is less than the effluent from the settling tank. The membrane can also separate the materials that cannot be settled in the settling tank from effluent. It is cost effective as well when there is space limitation or the land is expensive.

Comparison of two hydraulic retention times of 24 and 18 hours for both reactors showed that COD and TPH removal efficiency at 24 hrs HRT is higher than 18 hours.

Results from tests of removing COD and TPH for various ratios of oily pollutant revealed that with the ratio of 0.6 at both HRTs, the amount of COD and TPH removal obtained while with ratios of higher than 0.6, this removal was reduced.

The highest removal efficiency of COD and TPH was 97.3% and 98.8%, respectively. These were obtained by the hybrid membrane bioreactor, with oily pollutant concentration of 700ppm , the ratio of TPH/COD molasses 0.6, at HRT of 24 hours.

The fouling is the major problem with membranes in separation processes. Nevertheless, RBC was used as a pre-treatment stage and the most of the wastewater was treated before entering into the membrane which results in the reduction of the fouling. Membrane fouling in this study took place after 120 hours from the beginning and after cleaning the membrane was reutilized. This is more than the time needed in previous studies [14].
